# Uptake and mitochondrial dysfunction of alpha-synuclein in human astrocytes, cortical neurons and fibroblasts

**DOI:** 10.1186/2047-9158-2-20

**Published:** 2013-10-04

**Authors:** Nady Braidy, Wei-Ping Gai, Ying Hua Xu, Perminder Sachdev, Gilles J Guillemin, Xing-Mai Jiang, J William O Ballard, Martin P Horan, Zhi Ming Fang, Beng H Chong, Daniel Kam Yin Chan

**Affiliations:** 1 Centre for Healthy Brain Ageing, School of Psychiatry, University of New South Wales, Sydney, Australia; 2 Aged Care and Rehabilitation, Bankstown-Lidcombe Hospital, Sydney, Australia; 3 Department of Human Physiology and Centre for Neuroscience, Flinders University School of Medicine, Adelaide, Australia; 4 Faculty of Medicine, University of New South Wales, Sydney, Australia; 5 Neuropsychiatric Institute, Prince of Wales Hospital, Sydney, Australia; 6 School of Medical Sciences, University of New South Wales, Sydney, Australia; 7 St Vincent’s Centre for Applied Medical Research, Sydney, Australia; 8 St George Clinical School, University of New South Wales and St George Hospital, Sydney, Australia; 9 School of Biotechnology and Biomolecular Sciences, University of New South Wales, Sydney, Australia; 10 Aged Care and Rehabilitation Unit, Bankstown-Lidcombe Hospital, Eldridge Road, Bankstown, 2031, NSW, Australia

**Keywords:** Alpha-synuclein, Neurons, Astrocytes, Fibroblasts, Mitochondria

## Abstract

The accumulation and aggregation of alpha-synuclein (α-syn) in several tissue including the brain is a major pathological hallmark in Parkinson’s disease (PD). In this study, we show that α-syn can be taken up by primary human cortical neurons, astrocytes and skin-derived fibroblasts *in vitro*. Our findings that brain and peripheral cells exposed to α-syn can lead to impaired mitochondrial function, leading to cellular degeneration and cell death, provides additional evidence for the involvement of mitochondrial dysfunction as a mechanism of toxicity of α-syn in human cells.

## Introduction

Parkinson’s disease (PD) is the second most common neurodegenerative disorder after Alzheimer’s disease (AD) [1]. This debilitating degenerative movement disorder is characterized symptomatically by resting tremor, bradykinesia, muscle tone rigidity and impaired autonomic, sensory and cognitive function. These motor deficits are attributed to the selective loss of dopaminergic neurons projecting from the substantia nigra to the midbrain. Dystrophic neurons typically contain cytosolic protein aggregates (“Lewy bodies”) composed largely of the presynaptic protein alpha-synuclein [1]. While numerous neurotoxins and genes have been implicated in the development of PD, the exact aetiology of PD remains unclear [1-5]. Accumulative evidence suggests that α-syn may be involved in the initiation and disease progression [6,7]. Familial forms of PD are associated with either missense of gene mutations in the SNCA gene encoding for α-syn [8]. Mutations in the α-syn gene locus represents an important risk factor for PD in several genome-wide association studies using large cohorts worldwide [9].

The α-syn is a cytosolic protein that can be released from neuronal cells in small amounts via unconventional exocytosis under normal physiological conditions [10,11]. However, in the presence of several stressors, α-syn may be released from neuronal cells into the surrounding extracellular space in larger quantities and taken up by neighbouring cells by endocytosis [10,11]. However, little is known regarding the effect of human recombinant α-syn on other cells of the brain and surrounding tissue.

In this study, we have shown that human recombinant α-syn can be taken up by human primary astrocytes, cortical neurons and fibroblasts. We also show that this may lead to increased intracellular oxygen consumption and cell death after 24 hour exposure. The effect of α-syn *in vitro*, even in cultured fibroblasts, suggests that PD may be a systemic illness, with primary pathological changes occurring in the brain.

## Materials and methods

### Reagents and chemicals

Dulbecco’s phosphate buffer solution (DPBS) and all other cell culture media and supplements were from Invitrogen (Melbourne, Australia) unless otherwise stated. Mouse mAb anti-α-syn, DAPI, and pAb anti-GFAP were obtained from Sigma-Aldrich (Castle-Hill, Australia). Rabbit anti-MAP2 and anti-fibronectin were obtained from Millipore (Melbourne, Australia). Secondary anti-mouse IgG and anti-rabbit Alexa 488 (green) or Alexa 594 (red)-conjugated antibodies were purchased from Molecular Probes (Eugene, OR). All commercial antibodies were used at the concentrations specified by the manufacturers.

### Cell cultures

Human foetal brains, and skin were obtained from 16–19 week old foetuses collected following therapeutic termination with informed consent. Mixed brain cultures were prepared and maintained using a protocol previously described [12].

Astrocytes were prepared from the mixed brain cell cultures using a protocol previously described [13]. Cells were cultured in medium RPMI 1640 (Invitrogen) supplemented with 10% foetal bovine serum, 1% 1-glutamax, 1% antibacterial/antifungal, and 0.5% glucose. Cells were seeded into 24-well tissue culture plates to a density of 1 × 10^5^ cells 24 hours prior to experimentation.

Human foetal cortical neurons were prepared from mixed brain cell cultures as previously described [14]. Briefly, cortical neurons were plated in 24-well culture plates coated with Matrigel (BD Biosciences) diluted 1/20 in Neurobasal (Invitrogen). The culture medium was changed every second day and consisted of Neurobasal medium supplemented with 1% B-27 supplement, 1% Glutamax, 1% antibiotic/antifungal, and 0.5% glucose.

Fibroblasts were prepared from the skin using a protocol as previously described [15]. All cell cultures were maintained in an incubator at 37°C in a humidified atmosphere containing 95% air/5% CO_2_.

### Uptake of recombinant extracellular α-synuclein

Recombinant α-synuclein was expressed in Escherichia coli BL21(DE3) transformed with the pET11d vector containing the human α-synuclein cDNA described previously [16]. For uptake of recombinant α-syn in primary astrocytes, cortical neurons and skin-derived fibroblasts, human α-syn was incubated overnight at 37°C to form oligomers and then labelled with Alexa Fluor 488 (Invitrogen) as per the manufacturer’s instructions. Cells were incubated with expansion media containing 1 μM Alexa-Fluor-488-labelled α-syn for 30 min and 60 min. Cells were washed twice with PBS to ensure that the uptake of exogenous α-syn was not due to non-specific binding of α-syn to the cells.Cells were fixed with 4% paraformaldehyde diluted in DPBS.

### Immunocytochemistry for the detection of α-syn uptake

The method for immunocytochemistry has been previously described [17]. Cells were incubated with selected primary antibody mAb α-syn, together with phenotypic markers (GFAP, MAP-2, Fibronectin) and antibody against the inner mitochondrial membrane protein, complex V (ATPase) (Abcam). Selected secondary antibodies (goat anti-mouse IgG or goat anti-rabbit coupled with Alexa 488 or Alexa 594) were used. The following controls were performed for each labelled experiment: (1) isotypic antibody controls; and (2) incubation with only the secondary labelled antibody.

### Extracellular LDH activity as a measurement for cytotoxicity

The release of lactate dehydrogenase (LDH) into culture supernatant correlates with the amount of cell death and membrane damage, providing an accurate measure of cellular toxicity. LDH activity was assayed on cortical neurons, astrocytes and skin-derived fibroblasts treated with 5 μM recombinant α-syn after 24 hours incubation using a standard spectrophotometric technique described by [18].

### XF24 Microplate-based respirometry

To determine the effect of exogenous recombinant α-syn (5 μM) on oxygen consumption rates (OCRs; as indicator of mitochondrial respiration) in cortical neurons, primary astrocytes, and skin-derived fibroblasts, the Seahorse XF24, extracellular flux analyzer (Seahorse Bioscience, North Billerica, MA, USA) was employed as previously described. Briefly, culture plates were incubated in a CO_2_-free incubator at 37°C for 1 hr to equilibrate for temperature and pH. The microplate was then loaded into the XF24 and further incubated for 15 min by 3 min mix, 2 min wait cycles before commencement of the assay. The XF assay was performed as previous described [19]. After determination of the basal respiration in the cell culture, oligomycin (2 μM), carbonylcyanide-p-trifluoromethoxy-phenylhydrazone (FCCP, 500 nM), and antimycin (3 μM) were sequentially added and the oxygen consumption rates (OCRs) for each culture well were quantified for 2 minutes. This allowed us to determine the basal control ratio (BCR) and the uncoupling ratio (UCR) [20]. Essentially, the BCR is a measurement of how close the basal level of respiration is to the maximum level of respiration (i.e., basal/maximum). The closer this ratio is to 1, the lower the maximum level of respiration, and thus represents an indication of mitochondrial malfunction. The UCR is a measurement of mitochondrial functional integrity and measures the ratio of uncoupled to physiologically normal respiration levels (i.e., maximum/basal). The greater the maximum level of respiration, the greater the mitochondrial functional integrity.

### Bradford protein assay for the quantification of total protein

Extracellular LDH activity, NAD^+^ concentration, nuclei and mitochondrial protein content, and mitochondrial function were corrected for variations in protein concentration using the Bradford protein assay described by Bradford [21].

### Data analysis

Results obtained are presented as the means ± the standard error of measurement (SEM). One way analysis of variance (ANOVA) and post hoc Tukey’s multiple comparison tests were used to determine statistical significance between treatment groups. Differences between treatment groups were considered significant if p was less than 0.05 (p < 0.05). Experiments were performed in triplicates using cells derived from three different specimens unless otherwise stated.

## Results

### Time dependent uptake of α-syn in cultured human cortical neurons, astrocytes and skin-derived fibroblasts

**Figure 1:**
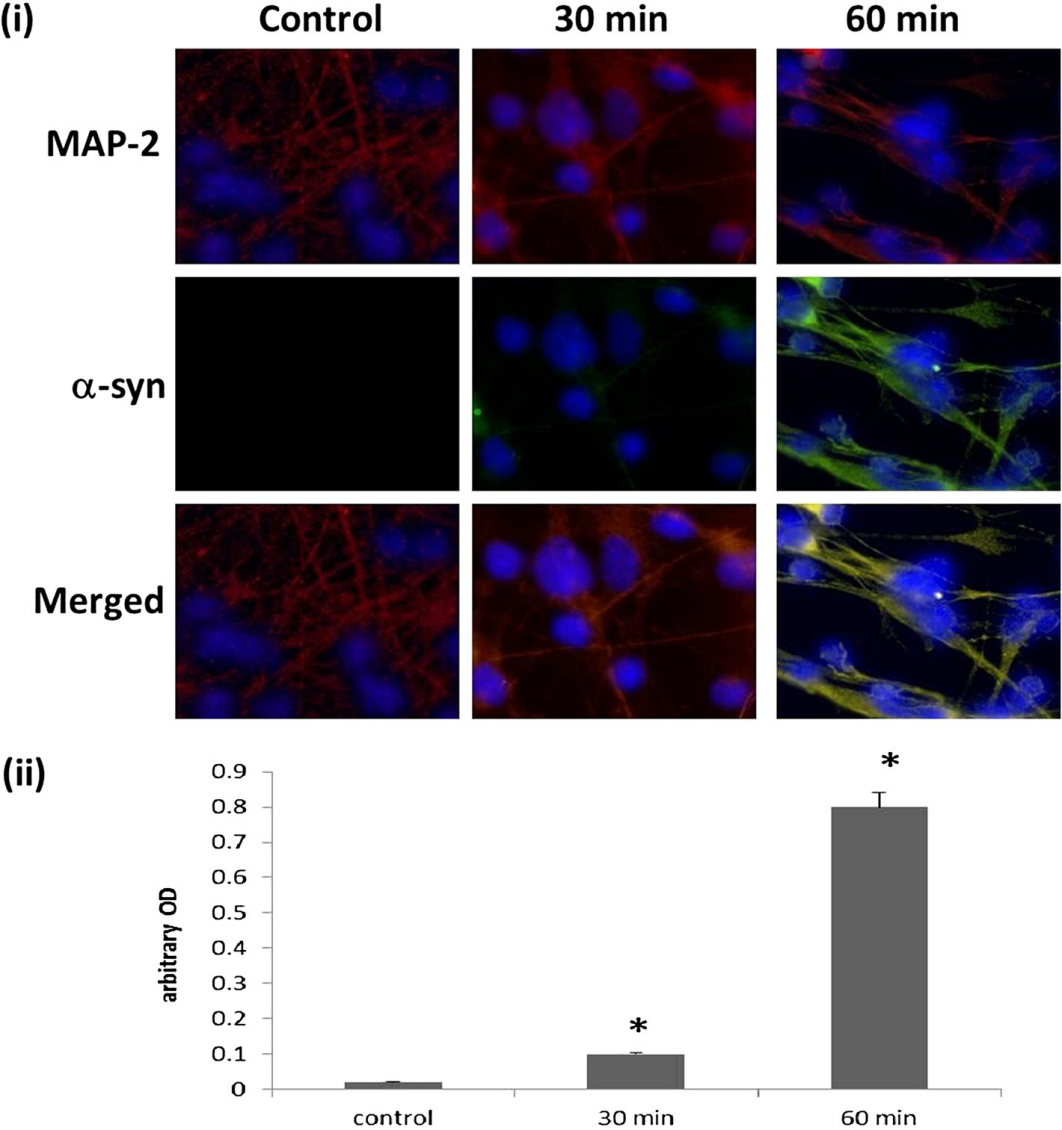
**α-syn uptake in human primary cortical neurons cells. (i)** Confocal microscopy analysis of α-syn uptake after 30 and 60 min in human cortical neurons. Staining for Alexa-Fluor-488-labelled α-syn in human neurons: Top, double staining for MAP-2/red and DAPI/blue; Centre, double staining for α-syn/green and DAPI/blue; Bottom, Merged MAP-2/red, α-syn/green and DAPI/blue.** (ii)** The histogram represents the semi-quantification of the immunostaining intensity. Intensity has been quantified using ImageJ 10.2. This experiment has been performed in triplicate with primary cultures of cortical neurons prepared from three different brain samples. Three individual microscopic fields were analysed for each treatment and the SEM for the data was determined to be 5%. * indicate the level of significance (p < 0.05).

**Figure 2:**
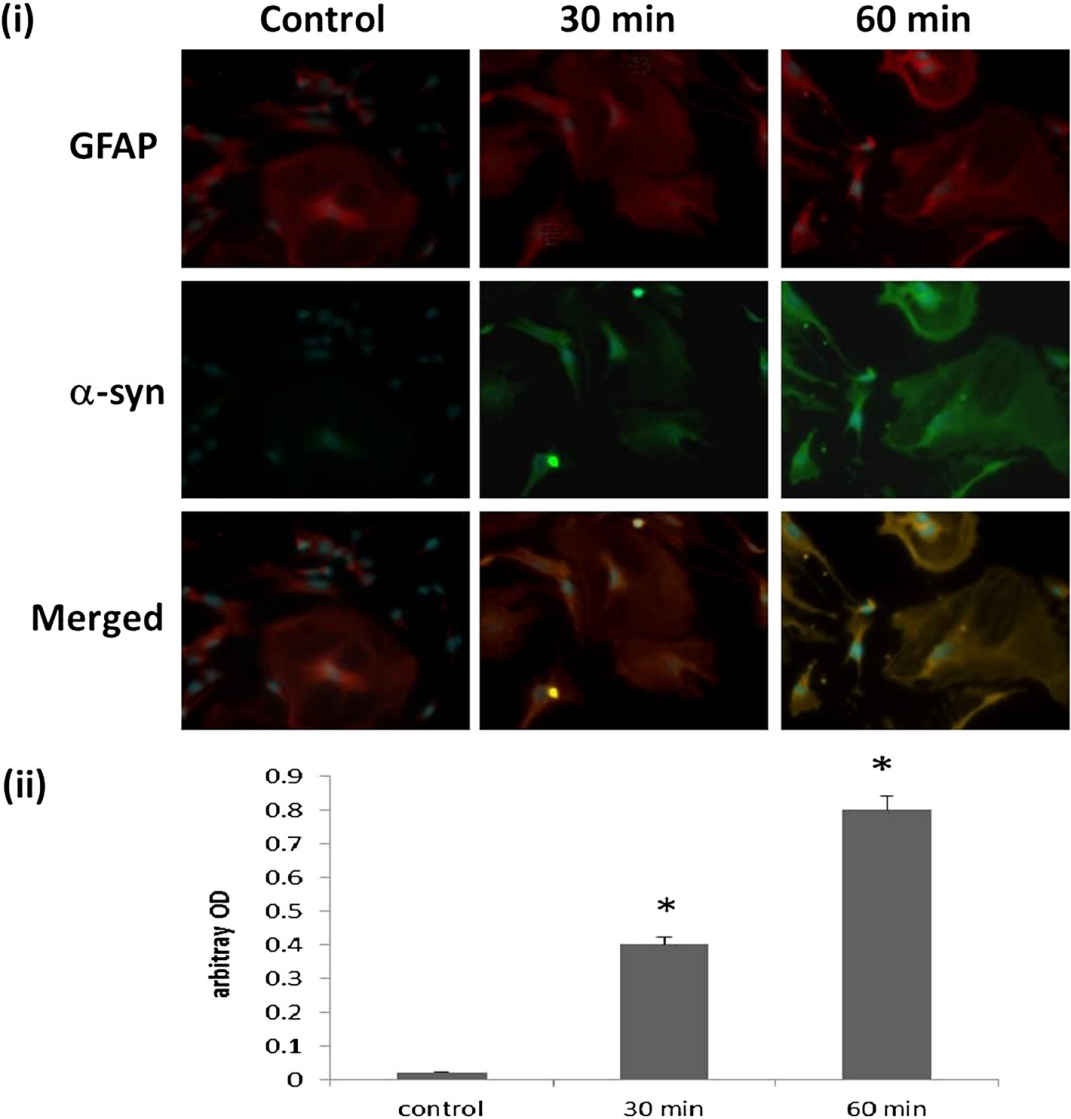
**α-syn uptake in human primary astrocytes. (i)** Confocal microscopy analysis of α-syn uptake after 30 and 60 min in human astrocytes. Staining for Alexa-Fluor-488-labelled α-syn in human neurons: Top, double staining for GFAP/red and DAPI/blue; Centre, double staining for α-syn/green and DAPI/blue; Bottom, Merged GFAP/red, α-syn/green and DAPI/blue. **(ii)** The histogram represents the semi-quantification of the immunostaining intensity. Intensity has been quantified using ImageJ 10.2. This experiment has been performed in triplicate with primary cultures of astrocytes prepared from three different brain samples. Three individual microscopic fields were analysed for each treatment and the SEM for the data was determined to be 5%. * indicate the level of significance (p < 0.05).

**Figure 3:**
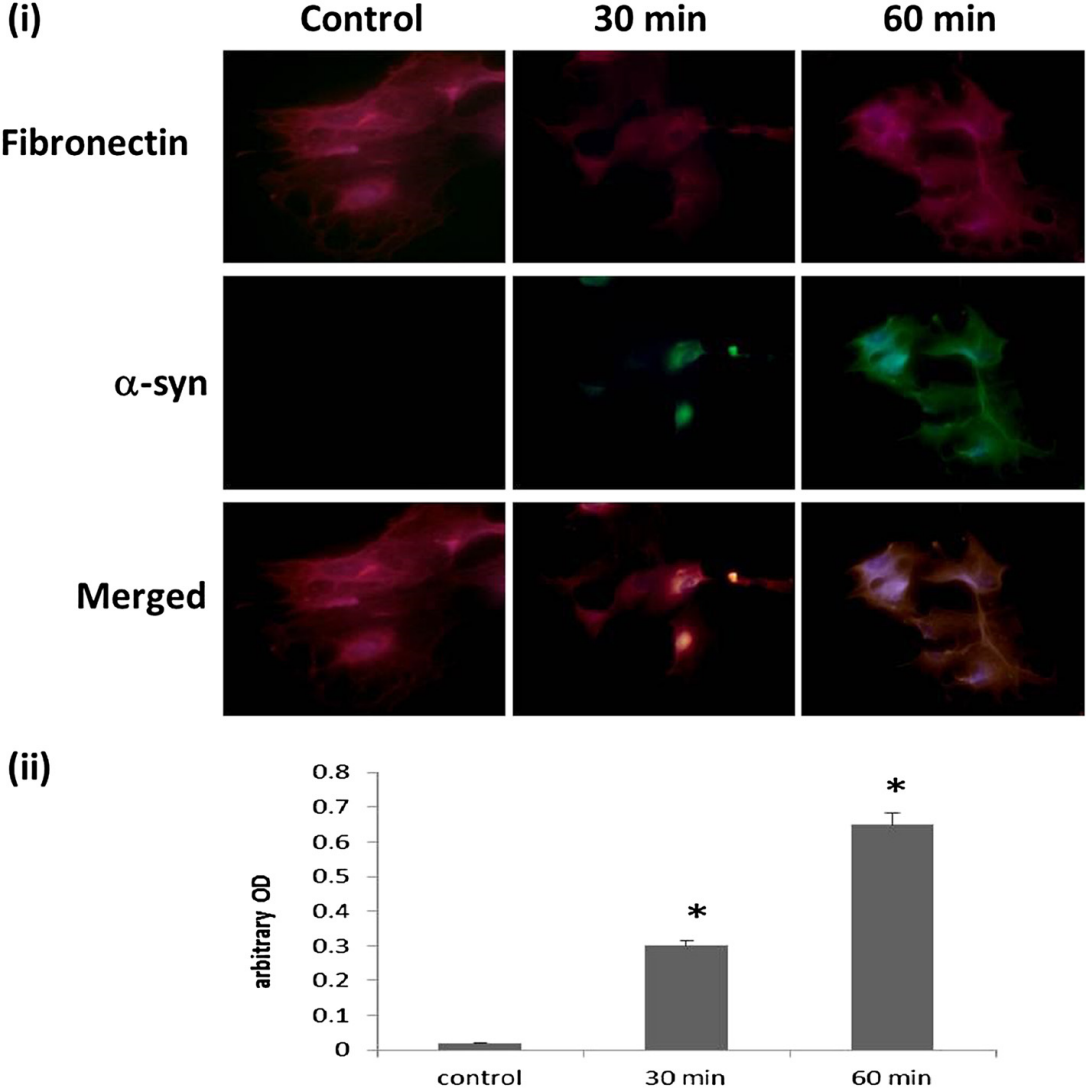
**α-syn uptake in human primary skin derived fibroblasts. (i)** Confocal microscopy analysis of α-syn uptake after 30 and 60 min in human skin-derived fibroblasts. Staining for Alexa-Fluor-488-labelled α-syn in human neurons: Top, double staining for Fibronectin/red and DAPI/blue; Centre, double staining for α-syn/green and DAPI/blue; Bottom, Merged Fibronectin /red, α-syn/green and DAPI/blue. **(ii)** The histogram represents the semi-quantification of the immunostaining intensity. Intensity has been quantified using ImageJ 10.2. This experiment has been performed in triplicate with primary cultures of fibroblasts prepared from three different fetal skin samples. Three individual microscopic fields were analysed for each treatment and the SEM for the data was determined to be 5%. * indicate the level of significance (p < 0.05).

To reaffirm the uptake of extracellular α-syn aggregates in human astrocytes, cortical neurons and fibroblasts, primary cultures were incubated with cell culture media containing Alexa-Fluor-488 labelled α-syn. Immunofluorescence analysis displayed in this study have highlighted the potential ability of these cell to uptake α-syn in a time-dependent manner (Figures 1, 2 and 3).

### Cytotoxic effects of α-syn in enteric neurons is associated increased intracellular oxygen consumption leading to cell death

**Figure 4:**
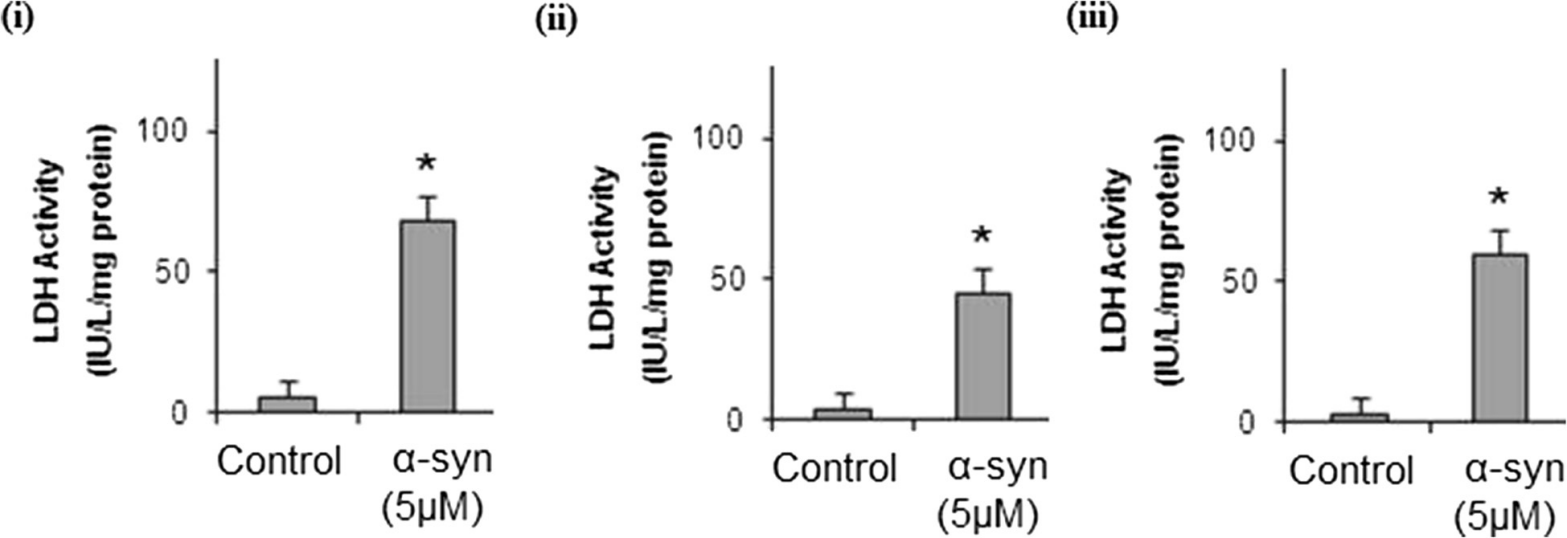
**Effects of α-syn on mitochondrial function in human primary cortical neurons, astrocytes and skin-derived fibroblasts.** The BCR and UCR were determined in **(i)** human primary cortical neurons, **(ii)** astrocytes and **(iii)** skin-derived fibroblasts pre-treated with 5 μM recombinant α-syn for 24 hours. Significance ^*^p < 0.05 compared to non-treated cells. (n = 4 for each treatment group).

**Figure 5:**
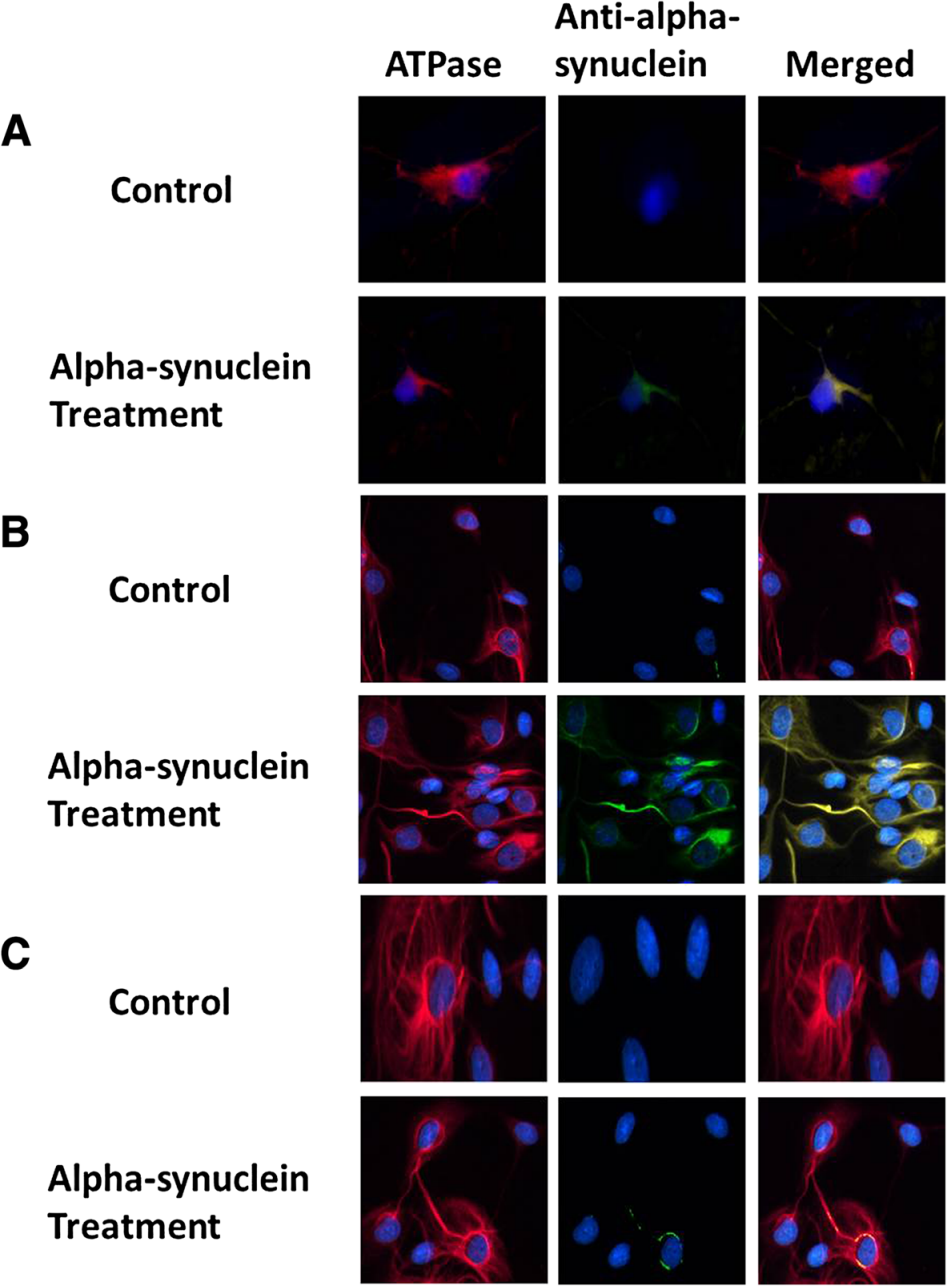
**Localisation of recombinant α-syn to the mitochondria in human primary (A) cortical neurons (B) astrocytes, and (C) skin derived fibroblasts.** Confocal microscopy analysis of α-syn uptake after 24 hours. Dual immunocytochemical staining for α-syn using the anti-alpha-syn antibody (R & D systems, Minneapolis, MN) (green) and inner mitochondrial membrane protein, complex V (ATPase, red).

**Figure 6:**
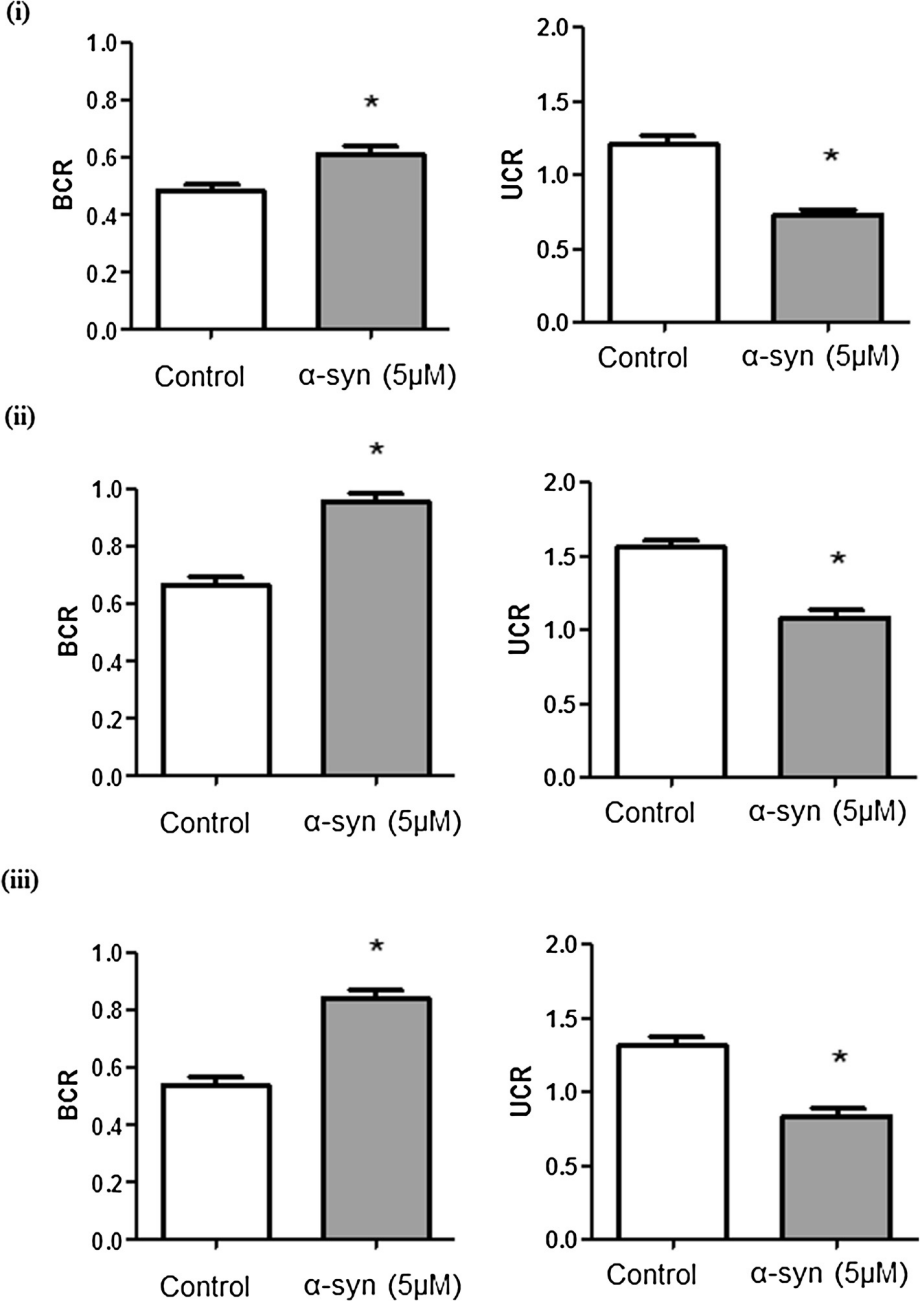
**Effects of α-syn on cell viability in human primary cortical neurons, astrocytes and skin-derived fibroblasts.** Effect of recombinant α-syn (5 μM) on extracellular LDH activity in **(i)** human primary cortical neurons, **(ii)** astrocytes and **(iii)** skin-derived fibroblasts pre-treated with 5 μM recombinant α-syn for 24 hours. ^*^p < 0.05 compared to non-treated cells (control); (n = 4 for each treatment group).

A recently study showed that recombinant extracellular α-syn forms cytoplasmic bodies capable of inducing toxicity at 5 μM after 24 hours incubation in SH-SY5Y neuroblastoma cell line [22]. Using the same dose and exposure time, we investigated the cytotoxic effect of recombinant α-syn in human astrocytes, cortical neurons and fibroblasts. A significant increase in basal control ratio (BCR) and a decline in uncoupling ratio (UCR) was observed after 24 hours incubation when these cells were treated with medium containing recombinant α-syn using the Seahorse XF24 (Seahorse Bioscience) (Figure 4). We further confirmed that α-syn is cytotoxic to the mitochondria by demonstrating that exogenous α-syn is localised in the mitochondria of the host cell (Figure 5). Increased extracellular LDH activity was also observed in these cells after 24 hours (Figure 6).

## Discussion

The current study uses several cell culture models: 1) to reaffirm whether recombinant α-syn can be taken up by primary human astrocytes, cortical neurons and fibroblasts; 2) to determine the effect of α-syn on mitochondrial function in brain and periphery cells using Seahorse XF240. A key finding of the current investigation is that α-syn can accumulate in several cell types *in vitro* not restricted to the brain, in a time dependent manner leading to increased intracellular oxygen consumption and cell death.

Owing to the diverse clinical symptoms associated with PD, it is likely that multiple brain systems may be impaired. Indeed, Lewy bodies are widely distributed in the several areas of the brain, including the midbrain, cerebrum and brainstem corresponding to axonal projections [23]. Earlier studies have suggested that misfolded α-syn may propagate from neuron to neuron in a prion-like manner, causing endogenous α-syn to misfold [11]. Recently, Freundt et al. [24] showed that fibrillar α-synuclein can be internalized by primary mouse neurons via rapid axonal transport with saltatory movement [24]. Moreover, Angot et al. [25] showed that α-syn can be transferred between neuronal host cells and grafted dopaminergic neurons *in vivo*[25]. However, our data is the first to demonstrate the uptake of exogenous α-syn oligomers in human primary cortical neurons. Taken together, these results support the hypothesis that PD can be caused by neuron-to-neuron spread of α-syn aggregates involving axonal transport of fibrillar and/or oligomers.

Glial inclusions, which are common pathological features in PD, have been shown to occur as a result of neuron-to-glia transmission of α-syn protein [26]. Here, we show that human primary astrocytes can also uptake α-syn oligomers similar to murine glial cells. One study showed that glial accumulation of α-syn proteins released from murine neuronal cells may involve endocytosis leading to the formation of cytoplasmic inclusion bodies [27]. Furthermore, astrocytes exposed to neuronal α-syn exhibited significant changes in their gene expression profile providing biochemical evidence for an inflammatory response. Up-regulation of pro-inflammatory cytokines and chemokines correlated positively with the degree of accumulation of α-syn in glial cells [26]. Together, these results suggest that astroglial alpha-synuclein pathology is produced by direct transmission of neuronal alpha-synuclein aggregates, causing inflammatory responses. This transmission step is thus an important mediator of pathogenic glial responses and could qualify as a new therapeutic target.

Our data is also in line with previous studies showing increased α-syn expression in Parkinson patient fibroblasts parallel to increased oxidative stress in these cells [28]. Without an appropriate mechanism of uptake into these distinct cell types, these observed patterns cannot be fully evaluated. They do, however, suggest that α-syn toxicity may not be restricted to the central and peripheral nervous system but may also be associated with other systems such as the skin. For example, it might be reasonable to hypothesise that α-syn aggregates can progress from neurons, and dissociate to interact with other cells, and vice versa, hence leading to a reduction in function which is not restricted to neuronal cells.

Mitochondrial dysfunction has been frequently documented in the neurodegenerative process that underlies PD [10,28-35]. Despite this, the molecular events leading to the observed dysfunction remains unclear. The goal of this study was also to investigate the effects of recombinant α-syn cell viability and mitochondrial function in primary human neurons, astrocytes and fibroblasts. Our data shows a significant increase in lactate dehydrogenase (LDH) release and indicating a reduction in cell viability in α-syn treated cells compared to non-treated controls. With regards to mitochondrial function, α-syn exhibited a significant reduction in oxygen consumption in these cells, as observed by an increase in the BCR ratio and a decline in the UCR. Importantly, these data also support a previous PD respiratory analysis study which found that α-syn over-expression also reduced the mitochondrial maximum respiration rate and increased mitochondrial fragmentation [34].

While α-syn is a well known cytoplasmic protein, numerous studies have shown that it has some affinity to phospholipids, and vesicles [36,37]. Herein we have shown that α-syn is also delivered to the mitochondria. Another study has shown that α-syn is present in nanomolar concentrations in neuronal culture medium [38]. Secreted forms of α-syn have been shown to re-enter adjacent cells leading to increased cytotoxicity via oxidative stress mechanisms [22,25]. In the present study, we showed that, in cultured neurons, glial cells and skin-derived fibroblasts, α-syn could form intracellular inclusions. Our study does not rule out the possibility that the internalized recombinant α-syn may be entangled with endogenous α-syn, and that the α-syn expression visualised herein may consist of both naturally occurring endogenous and recombinant exogenous α-syn. However, using our method, it is clear that endogenous α-syn is only expressed at very low levels in foetal tissue, and not detected using immunohistochemistry. Further work is warranted to investigate the binding properties of α-syn in cultured human cells.

The current data is consistent with mitochondrial dysfunction as the converging pathway connecting a variety of causes for PD. In fact, several causes of mitochondrial dysfunction due to free radicals and lysosomal/proteasome dysfunction associated with α-syn have been reported in PD [28,39,40]. One study showed a higher frequency in somatic mitochondrial DNA deletion in the substantia nigra of sporadic PD patients leading to deficiency in the respiratory chain and increased generation of reactive oxygen species [41]. These results are in line with our current work indicating that PD may occur in response to mitochondrial dysfunction, possibly due to alterations in gene expression.

## Competing interest

The authors declare that they have no competing interest.

## Authors’ contributions

Conceived and designed the experiments: NB and DKYC. Analyzed the data: NB, YHX, MPH and ZMF. Wrote the first draft of the manuscript: NB. Contributed to the writing of the manuscript: NB, YHX, MPH, PS, GJG and DKYC. Agree with manuscript results and conclusions: NB, YHX, MPH, PS, GJG, BHC, X-MJ, JWOB and DKYC. Made critical revisions and approved final version: NB, YHX, DKYC. All authors read and approved the final manuscript.
